# Supplementary material for: NIR-II fluorescence microscopic imaging of cortical vasculature in non-human primates
Source: Theranostics. 2020 Mar 4;10(9):4265–76. doi: 10.7150/thno.43533 (PMC7086344; doi:10.7150/thno.43533)
Supplement: Supplementary file 1 — Supplementary figures and movie legends. [file thnov10p4265s1.pdf]

NIR-II fluorescence microscopic imaging of cortical vasculature in non-human primates

Zhaochong Cai[1,#], Liang Zhu[2#], Mengqi Wang[2], Anna Wang Roe[3,4,5]*, Wang Xi[3,4]*, Jun Qian[1]*

[#]These authors contributed equally

*Corresponding authors: qianjun@zju.edu.cn (Jun Qian); xw333@zju.edu.cn (Wang Xi); annawang@zju.edu.cn (Anna Wang Roe)

[1]State Key Laboratory of Modern Optical Instrumentations, Centre for Optical and Electromagnetic Research, College of Optical Science and Engineering, Zhejiang University, Hangzhou 310058, China.

[2]Interdisciplinary Institute of Neuroscience and Technology (ZIINT), College of Biomedical Engineering and Instrument Science, Zhejiang University, Hangzhou 310027, China.

[3]Interdisciplinary Institute of Neuroscience and Technology (ZIINT), the Second Affiliated Hospital, School of Medicine, Zhejiang University, Hangzhou 310020, China.

[4]Key Laboratory of Biomedical Engineering of Ministry of Education, College of Biomedical Engineering and Instrument Science, Zhejiang University, Hangzhou 310027, China.

[5]Division of Neuroscience, Oregon National Primate Research Center, Oregon Health and Science University, Beaverton, OR 97239, USA.

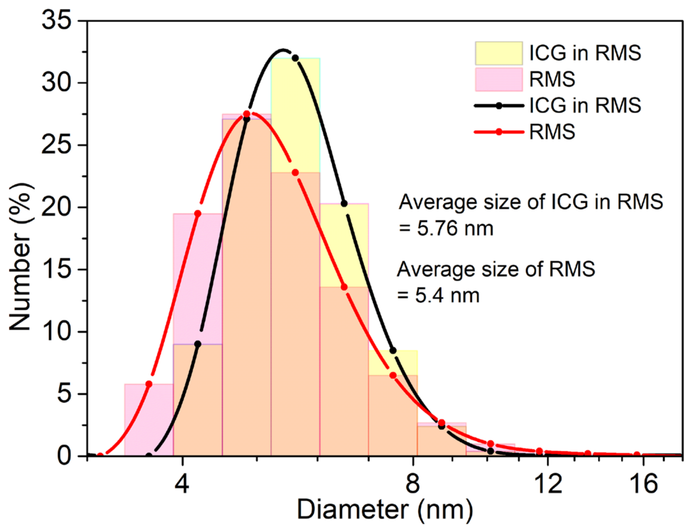

Supplementary Figure 1. Hydrodynamic mean size distributions of rhesus macaque serum (pink) and ICG in rhesus macaque serum (yellow) by dynamic light scattering. These distributions are significantly different (RMS mean: 5.4 nm, ICG in RMS mean: 5.76 nm; ($X^2 = 131.6$, $p < 0.001$).

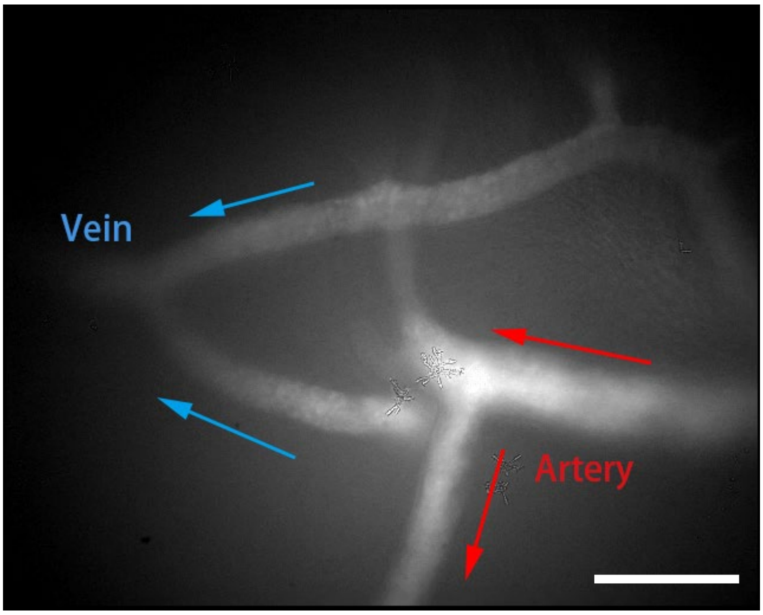

Supplementary Figure 2. NIR-II fluorescence wide-field microscopic image of cerebral vessels showing the blood flow directions and determination of arteries and veins. Depth = 180 μm. Scale bar: 100 μm.

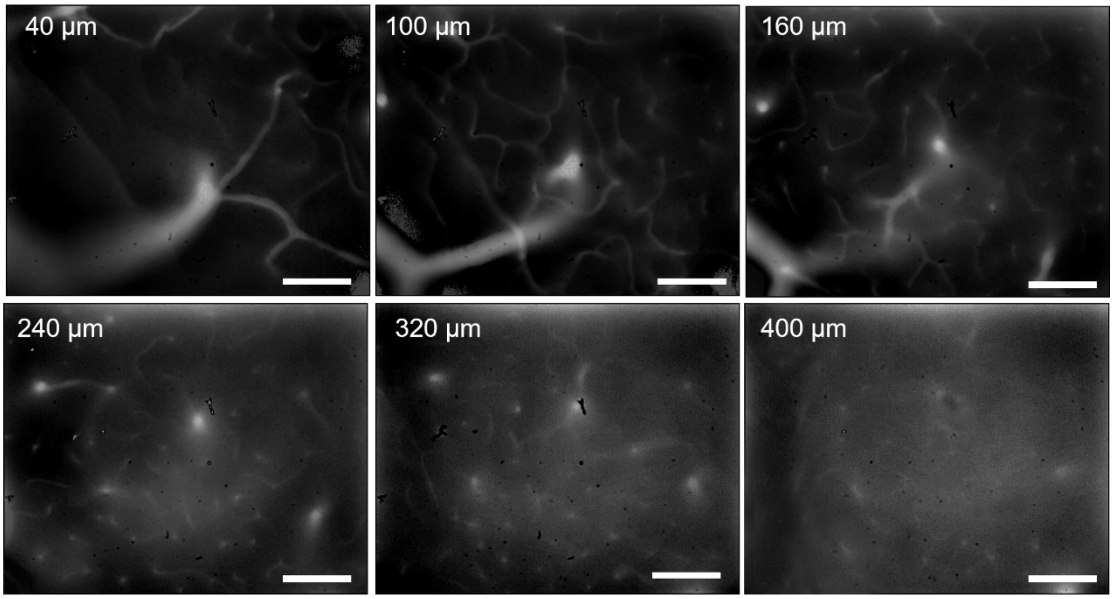

Supplementary Figure 3. NIR-II fluorescence wide-field microscopic images of cerebral blood vessels of the rhesus macaque at various depths (40 μm, 100 μm, 160 μm, 240 μm, 320 μm, and 400 μm). Scale bars: 100 μm.

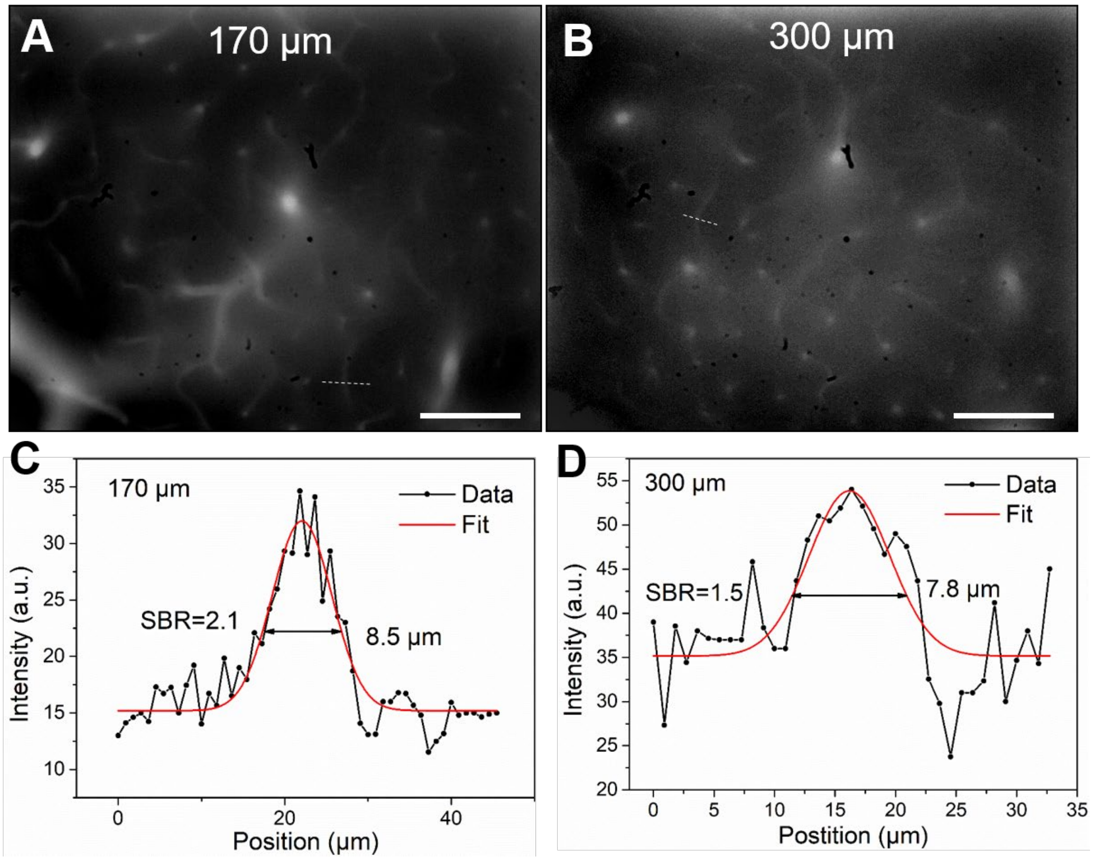

Supplementary Figure 4. A and **B** NIR-II fluorescence wide-field microscopic images of cerebral blood vessels of the rhesus macaque at two typical depths (170 μm and 300 μm). **C-D** The cross-sectional fluorescence intensity profiles (black) and the related Gaussian fitting (red) along the capillaries indicated by the white-dashed lines in **A** and **B**. Scale bars: 100 μm.

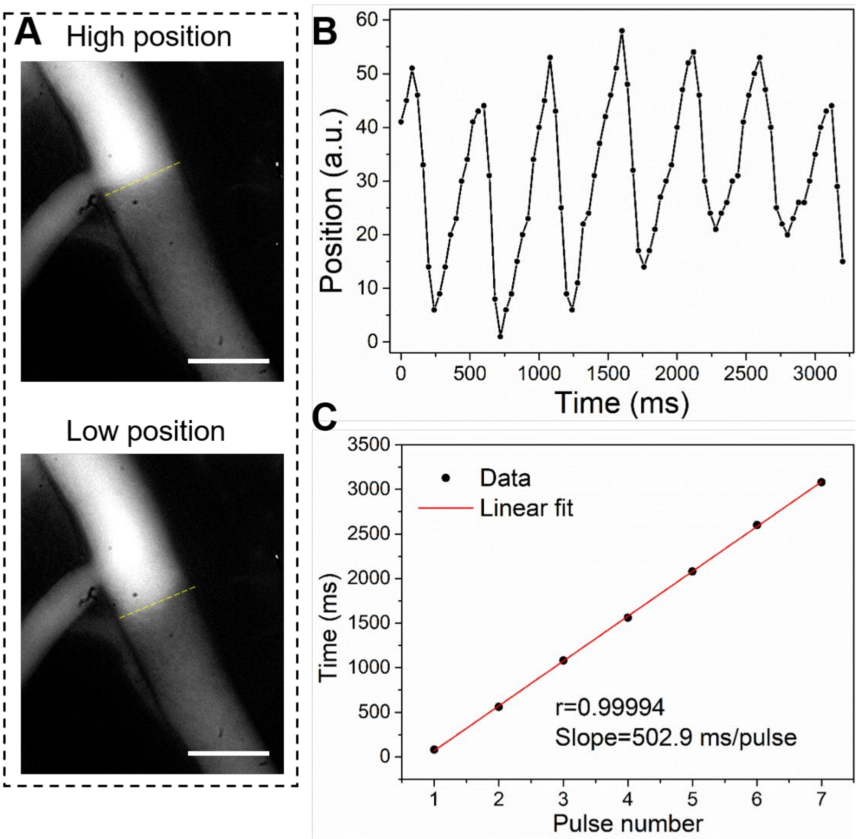

Supplementary Figure 5. Measurement of cardiac impulse period of the rhesus macaque based on NIR-II fluorescence wide-field microscopic brain vascular imaging. **A** Two typical images with the fluorescence bright/dark border in the highest (top image) and lowest (bottom image) position in one cerebral blood vessel. Depth = 130 µm. Scale bars: 100 µm. **B** A plot of the position of the bright/dark boundary in the vessel as a function of time. **C** A plot of the peak timepoints of each impulse shown in **B**. The linear fit reveals an average cardiac impulse period of 502.9 ms/pulse, matching the 120 pulses/minute recorded on the heart rate monitor.

NIR-II fluorescence confocal microscopy in mice.

Prior to using the NIR-II fluorescence confocal microscope in primates, we first evaluated its performance in mice. As shown in Supplementary Fig. 6 and Supplementary Fig. 7, we imaged cerebral vasculature through a cranial window on a mouse which had received an intravenous injection of ICG (1 mg/mL, 200 μL). Images at various depths were obtained and a 3D image was reconstructed, revealing a clear vascular network (Supplementary Fig. 6). In addition, the spatial resolution and SBR were analyzed (Supplementary Fig. 7). This verified the superior imaging performance of our confocal setup.

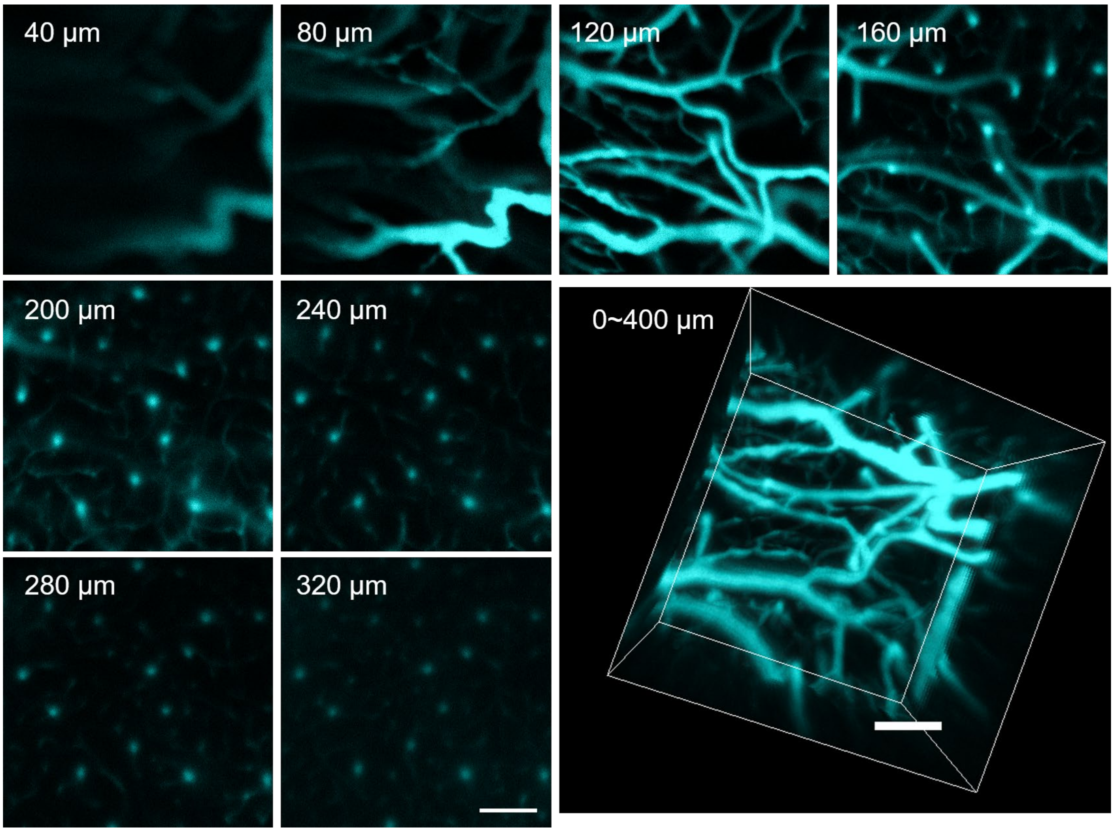

Supplementary Figure 6. NIR-II fluorescence confocal microscopic images of cerebral blood vessels of mouse at various depths and a 3D reconstructed image over depths of 0-400 μm. Scale bars: 100 μm.

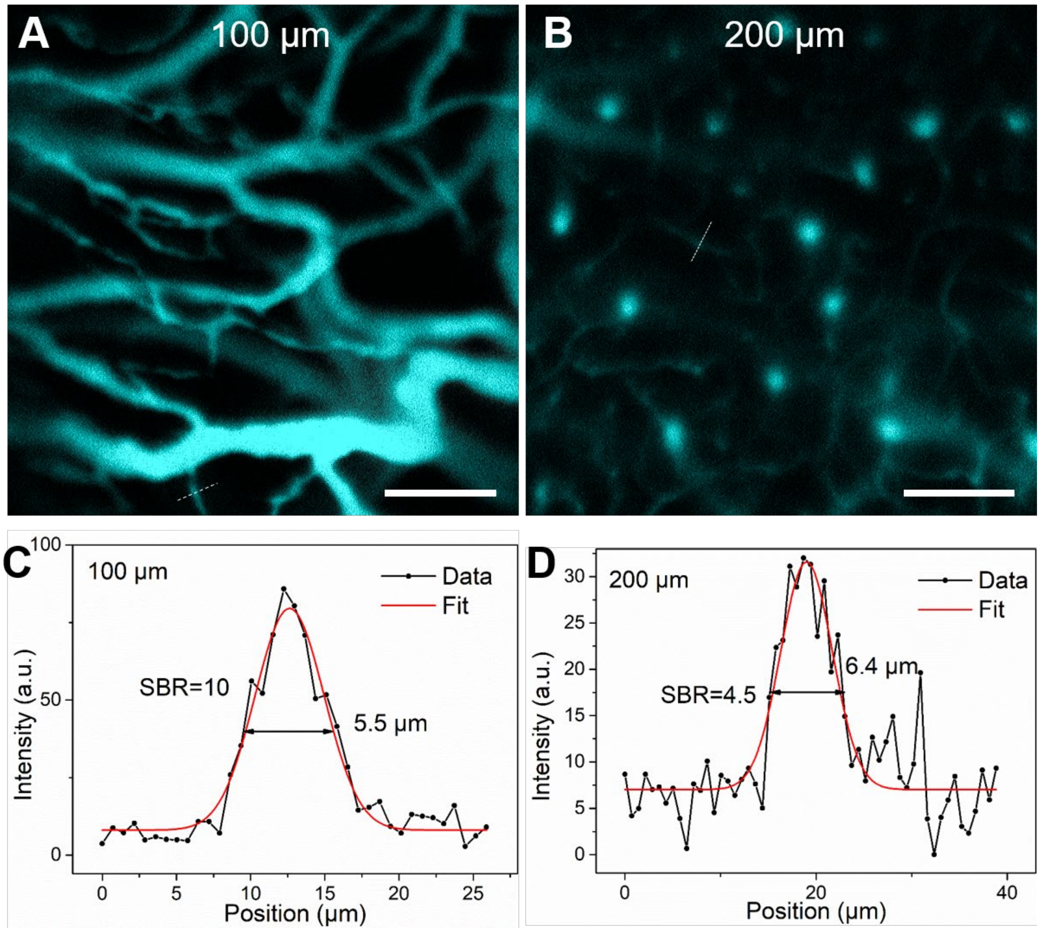

Supplementary Figure 7. A and **B** NIR-II fluorescence confocal microscopic images of cerebral blood vessels of the mouse at two typical depths (100 μm and 200 μm). **C-D** The cross-sectional fluorescence intensity profiles (black) and the related Gaussian fitting (red) along the capillary vessels indicated by the white-dashed lines in **A** and **B**. Scale bars: 100 μm.

MOV S1. A movie showing the NIR-II fluorescence wide-field microscopic *in vivo* imaging the flow of cerebral blood vessels in the rhesus macaque, with the objective magnification of 25×.

MOV S2. NIR-II fluorescence wide-field microscopic *in vivo* imaging showing the blood flow directions in cerebral vessels, as well as the discrimination of artery and vein.

MOV S3. NIR-II fluorescence wide-field microscopic *in vivo* imaging showing the moving of fluorescence bright/dark border in one cerebral blood vessel acompanied with the cardiac impulse.

MOV S4. 3D reconstructed NIR-II fluorescence confocal microscopic *in vivo* images of cerebral blood vessels of the rhesus macaque.
